# Reimbursement Legislations and Decision Making for Orphan Drugs in Central and Eastern European Countries

**DOI:** 10.3389/fphar.2019.00487

**Published:** 2019-05-08

**Authors:** Krzysztof Piotr Malinowski, Paweł Kawalec, Wojciech Trąbka, Marcin Czech, Guenka Petrova, Manoela Manova, Alexandra Savova, Pero Draganić, Lenka Vostalová, Juraj Slabý, Agnes Männik, Kristóf Márky, Zinta Rugaja, Jolanta Gulbinovic, Tomas Tesar, Marian Sorin Paveliu

**Affiliations:** ^1^Institute of Public Health, Faculty of Health Sciences, Jagiellonian University Medical College, Kraków, Poland; ^2^Bioinformatics and Public Health Department, Faculty of Medicine and Health Sciences, Andrzej Frycz Modrzewski Krakow University, Kraków, Poland; ^3^Department of Pharmacoeconomics, Institute of Mother and Child, Warsaw, Poland; ^4^Faculty of Pharmacy, Medical University of Sofia, Sofia, Bulgaria; ^5^National Council on Prices and Reimbursement of Medicinal Products, Sofia, Bulgaria; ^6^Agency for Medicinal Products and Medical Devices of Croatia, Zagreb, Croatia; ^7^Department of Biotechnology, University of Rijeka, Rijeka, Croatia; ^8^Pricing and Reimbursement Regulation Branch, State Institute for Drug Control, Prague, Czechia; ^9^Institute of Family Medicine and Public Health, University of Tartu, Tartu, Estonia; ^10^National Institute of Health Insurance Fund Management, Budapest, Hungary; ^11^The National Health Service, Riga, Latvia; ^12^Department of Pathology, Forensic Medicine and Pharmacology, Institute of Biomedical Sciences, Faculty of Medicine, Vilnius University, Vilnius, Lithuania; ^13^Department of Organisation and Management in Pharmacy, Faculty of Pharmacy, Comenius University in Bratislava, Bratislava, Slovakia; ^14^Titu Maiorescu University, Bucharest, Romania

**Keywords:** orphan drug, reimbursement policy, Central and East Europe, European Medicine Agency, kappa coefficient of agreement, marketing authorisation, exceptional circumstances, conditional approval

## Abstract

**Background:**

Reimbursement policies influence access of patients to orphan drugs in the European countries.

**Objectives:**

To provide a comprehensive description of orphan drug reimbursement policies and to assess reimbursement decision-making process in the EU-CEE countries as well as the impact of the type of approval and disease on reimbursement decisions.

**Methods:**

For each drug, the information regarding conditional approval or approval under exceptional circumstances was obtained from the EMA website. The reimbursement status for analyzed drugs was collected in a questionnaire survey performed in a group of experts in reimbursement policy. The agreement between countries was assessed using the κ coefficient, nominal variables tests were compared using the χ2 test or the Fisher exact test. The impact of the EMA’s conditional approval and approval under exceptional circumstances was assessed using logistic regression and presented as an odds ratio (OR).

**Results:**

The analysis revealed that most orphan drugs were authorized for the treatment of oncological or metabolic diseases [36 drugs (38%) and 22 drugs (23%), respectively]. The shares of reimbursed orphan drugs varied significantly (*p* = 0.0031) from 6.3% in Latvia to 27.4% in Poland. No correlation (*r* = 0.02; *p* = 0.9583) with GDP per capita was observed. The highest agreement in reimbursement decisions was observed between Estonia and Lithuania, and the lowest – between Estonia and Latvia, with kappa of 0.69 and 0.11, respectively. Significant impact of the type of approval and reimbursement status was observed for Czechia, Lithuania and Slovakia where conditional approval and exceptional circumstances negatively influenced reimbursement decision. Type of disease has significant influence on reimbursement decision in 4 out of 10 analyzed countries with significant outweigh of positive decisions for oncological diseases.

**Conclusion:**

In considered countries specific regulations on reimbursement of orphan drugs are valid but in Lithuania and Romania no formal HTA process was employed; in case of some countries higher ICER values for orphans are used. The share of reimbursed orphan drugs varied significantly across the countries, but it was not associated with GDP per capita.

## Introduction

Rare diseases mostly include inherited life-threatening or chronically debilitating diseases that affect fewer than 5 out of 10,000 people, according to the definition issued by the European Medicines Agency (EMA), however, the definition can vary between countries. This results in an approximate number of 246,000 patients affected by rare diseases in 27 European Union (EU) member countries (European Medicines Agency, 2007; Winstone et al., 2015). Reimbursement of drugs for rare diseases (so-called orphan drugs – approved by centralized procedure) is the most important factor that can increase the accessibility of treatment for patients. The EMA provides three types of approval: (a) conditional – a temporary approval until more data from clinical trials are available and the conditions will be fulfilled; (b) exceptional circumstances – a status that indicates that it is not possible to obtain additional data; and (c) approval without additional conditions (European Commission, 2006; European Medicines Agency, 2018a). Unlike the conditional approval, in which a marketing approval is granted on condition that a sponsor will provide relevant data within an agreed time frame, authorization under exceptional circumstances can be granted even when the more precise data will not be available for a more comprehensive assessment - usually data are further collected via registries instead of clinical trials. In all situations, the benefit of the product should outweigh the risk.

Both conditional approval and exceptional circumstances could influence reimbursement decision-making and should be considered in reimbursement policies, especially in countries with a limited budget (European Commission, 2006), such as the member states that joined the EU in 2004 as mostly middle- and low-income countries from the Central Eastern European (CEE) region. The situation has gradually improved for these countries, as they have expanded their pharmaceutical shares of health spending at an 8-fold higher annual rate compared with the 15 original EU countries (EU15; Jakovljevic et al., 2016). Orphan drugs along with targeted biologics are considered the most expensive pharmaceuticals (Jakovljevic and Yamada, 2017); therefore, it is especially important to improve their availability in CEE countries. Proper allocation of public resources represents a major challenge for public health and health care decision-making and seems to be reflected in substantial differences in reimbursement decisions for orphan drugs among the EU15 countries (Malinowski et al., 2018).

There are different classes of orphan drugs, with the broadest classes including oncological drugs [around 32.5% of orphan drugs (Gammie et al., 2015)] and drugs for metabolic conditions.

Our objective of this study was to assess and compare the percentage of reimbursed orphan drugs as well as the agreement in reimbursement decision-making between selected CEE countries. We also aimed to evaluate if reimbursement decisions were influenced by whether the EMA granted conditional approval or approval under exceptional circumstances. The impact of the type of disease (oncological or metabolic) on the type of approval (conditional or under exceptional circumstances) was also examined. Overall, we aimed to provide a comprehensive review of reimbursement policies for orphan drugs in EU–CEE countries.

## Materials and Methods

For each drug, the information regarding conditional approval or approval under exceptional circumstances was obtained from the EMA website (European Medicines Agency, 2018b). The reimbursement status for selected drugs was collected in a questionnaire survey performed in a group of experts in reimbursement policy and orphan drugs in the following EU-CEE countries: Bulgaria, Croatia, Czechia, Estonia, Hungary, Latvia, Lithuania, Poland, Romania, and Slovakia in 2017–2018. Data on the reimbursement system and decision-making process in these countries were also collected. Additional analyses were performed for the relevant subgroups of drugs used in the treatment of patients with oncological or metabolic conditions. Finally, the results for these two subgroups of drugs were compared with drugs used for the treatment of patients with other diseases (neither oncological nor metabolic; European Medicines Agency, 2018b).

The agreement (share of agreed answers over the expected at random) between countries was assessed using the κ coefficient, with values lower than 0 denoting less than chance agreement; between 0.01 and 0.20, slight agreement; between 0.21 and 0.40, fair agreement; between 0.41 and 0.60, moderate agreement; between 0.61 and 0.80, substantial agreement; and between 0.81 and 0.99, almost perfect agreement (Viera and Garrett, 2005). All κ coefficients were supported with 95% confidence intervals (CIs) and rounded to 2 decimal places.

Two nominal variables were performed using the χ^2^ test or the Fisher exact test, as applicable, depending on expected cell counts in contingency tables. The results of the tests were presented as *p*-values rounded to 4 decimal places. The data were summarized with counts and percentages.

The impact of the EMA’s conditional approval and approval under exceptional circumstances was assessed using logistic regression and presented as an odds ratio (OR) showing the odds for reimbursement when these types of approval were granted compared with no conditional approval or approval under exceptional circumstances. Logistic regression was also used to investigate the impact of the type of disease on positive reimbursement decisions. All ORs were presented with 95% CIs rounded to 2 decimal places and with corresponding *p*-values rounded to 4 decimal places. A *p*-value of less than 0.05 was considered statistically significant. Statistical analyses were carried out in the JMP^®^ software, version 14.0.0 (SAS Institute Inc., 2018, Cary, NC, United States).

## Results

**FIGURE 1:**
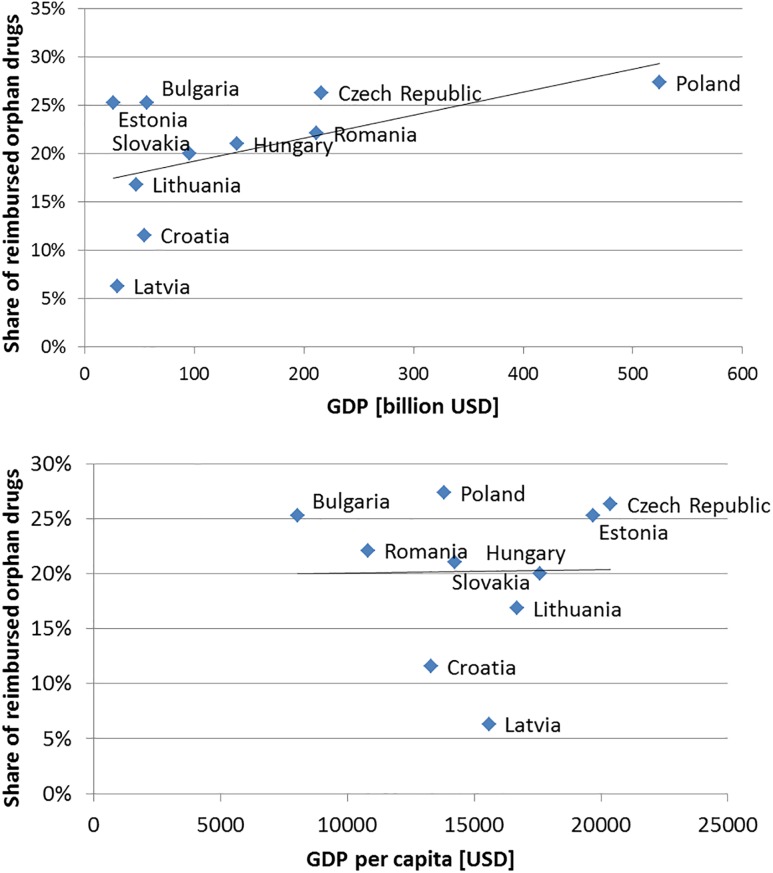
Relationship between share of reimbursed orphan drugs among analyzed countries and total gross domestic product (GDP) and GDP per capita with trend line.

The reimbursement status of 95 orphan drugs was assessed. The analysis revealed that most orphan drugs were authorized for the treatment of patients with oncological or metabolic diseases (36 drugs (38%) and 22 drugs (23%), respectively; Table 1]. The shares of reimbursed orphan drugs varied significantly (*p*-value of 0.0031) from a minimum of 6.3% in Latvia to a maximum of 27.4% in Poland (Figure 1). We observed that the shares of reimbursed orphan drugs experienced a trend with the total gross domestic product (GDP; correlation of 0.53), although the result was not significant (*p*-value of 0.1185). Additionally, no correlation (correlation of 0.02; *p*-value of 0.9583) was observed when analyzing GDP per capita (Figure 1).

**Table 1:** Reimbursement status of orphan drugs in 10 Central Eastern European Countries in 2017.

Medicine name	Drug type	Common name	Conditional approval	Exceptional circumstance	Bulgaria	Croatia	Czechia	Estonia	Hungary	Latvia	Lithuania	Poland	Romania	Slovakia
Adcetris	Oncological	Brentuximab vedotin	Yes	No	✓	✓	×	✓	✓	×	×	✓	×	×
Adempas	Other	Riociguat	No	No	×	×	✓	✓	×	×	✓	✓	✓	×
Alprolix	Other	Eftrenonacog alfa	No	No	×	×	×	✓	×	×	×	×	×	×
Arzerra	Oncological	Ofatumumab	No	No	✓	✓	×	×	×	×	×	×	✓	✓
Atriance	Oncological	Nelarabine	No	Yes	✓	×	×	×	✓	×	×	✓	✓	×
Blincyto	Oncological	Blinatumomab	Yes	No	×	×	×	×	×	×	×	×	×	×
Bosulif	Oncological	Bosutinib	Yes	No	✓	✓	×	✓	✓	✓	×	✓	✓	×
Bronchitol	Other	Mannitol	No	No	×	×	×	✓	×	×	×	×	×	×
Carbaglu	Metabolic	Carglumic acid	No	No	×	×	×	×	×	×	×	×	✓	×
Cayston	Other	Aztreonam	No	No	×	×	×	×	×	×	×	×	×	×
Ceplene	Oncological	Histamine dihydrochloride	No	Yes	×	×	×	×	×	×	×	×	×	×
Cerdelga	Metabolic	Eliglustat	No	No	×	×	×	×	×	×	×	×	×	×
Coagadex	Metabolic	Human coagulation factor X	No	No	×	×	×	×	×	×	×	×	×	×
Cometriq	Oncological	Cabozantinib	Yes	No	×	×	×	×	×	×	×	×	×	×
Cresemba	Other	Isavuconazole	No	No	×	×	×	×	×	×	×	×	×	×
Cystadane	Metabolic	Betaine anhydrous	No	No	×	×	×	×	×	×	×	✓	✓	×
Dacogen	Oncological	Decitabine	No	No	×	×	×	×	×	×	×	×	✓	×
Darzalex	Oncological	Daratumumab	Yes	No	×	×	×	×	×	×	×	×	×	×
Defitelio	Other	Defibrotide	No	Yes	×	×	×	×	×	×	×	×	×	×
Deltyba	Other	Delamanid	Yes	No	×	×	×	×	×	×	×	×	×	×
Diacomit	Other	Stiripentol	No	No	×	×	✓	✓	×	×	✓	✓	✓	×
Elaprase	Metabolic	Idursulfase	No	Yes	✓	✓	✓	×	×	×	×	✓	✓	×
Esbriet	Other	Pirfenidone	No	No	×	×	✓	✓	✓	×	✓	✓	×	×
Farydak	Oncological	Panobinostat	No	No	×	×	×	×	×	×	×	×	×	×
Firazyr	Other	Icatibant	No	No	×	×	✓	×	✓	×	×	✓	×	×
Firdapse (previously Zenas)	Other	Amifampridine	No	Yes	×	×	×	×	×	×	×	×	×	×
Galafold	Metabolic	Migalastat	No	No	×	×	×	×	×	×	×	×	×	×
Gazyvaro	Oncological	Obinutuzumab	No	No	×	✓	✓	×	✓	✓	✓	✓	×	✓
Gliolan	Oncological	5-aminolevulinic acid hydrochloride	No	No	×	×	×	×	×	×	×	×	×	×
Glybera	Metabolic	Alipogene tiparvovec	No	Yes	×	×	×	×	×	×	×	×	×	×
Granupas (previously para-aminosalicylic acid Lucane)	Other	Para-aminosalicylic acid	No	No	×	×	×	×	×	×	×	×	×	×
Hetlioz	Other	Tasimelteon	No	No	×	×	×	×	×	×	×	×	×	×
Holoclar	Other	*Ex vivo* expanded autologous human corneal epithelial cells containing stem cells	Yes	No	×	×	×	×	×	×	×	×	×	×
Iclusig	Oncological	Ponatinib	No	No	×	×	×	×	×	×	×	×	×	✓
Idelvion	Other	Albutrepenonacog alfa	No	No	×	×	×	×	×	×	×	×	×	×
Imbruvica	Oncological	Ibrutinib	No	No	✓	×	×	✓	✓	×	✓	✓	×	✓
Imnovid (previously pomalidomide Celgene)	Oncological	Pomalidomide	No	No	×	×	✓	×	×	×	×	×	×	×
Increlex	Metabolic	Mecasermin	No	Yes	×	×	✓	×	×	×	×	✓	×	×
Inovelon	Other	Rufinamide	No	No	×	×	✓	×	✓	×	×	×	×	✓
Kalydeco	Metabolic	Ivacaftor	No	No	×	×	×	×	×	×	×	×	×	×
Kanuma	Metabolic	Sebelipase alfa	No	No	×	×	×	×	×	×	×	×	×	×
Ketoconazole HRA	Metabolic	Ketoconazole	No	No	×	×	×	×	×	×	×	×	×	×
Kolbam	Metabolic	Cholic acid	No	Yes	×	×	×	×	×	×	×	×	×	×
Kuvan	Metabolic	Sapropterin	No	No	✓	×	✓	✓	×	×	×	×	✓	✓
Kyprolis	Oncological	Carfilzomib	No	No	×	×	×	×	×	×	×	×	×	×
Lartruvo	Oncological	Olaratumab	Yes	No	×	×	×	×	×	×	×	×	×	×
Lenvima	Oncological	Lenvatinib	No	No	×	×	×	×	×	×	×	×	×	×
Lynparza	Oncological	Olaparib	No	No	✓	✓	×	✓	×	×	✓	✓	×	×
Mepact	Oncological	Mifamurtide	No	No	×	×	✓	✓	×	×	×	×	×	×
Mozobil	Other	Plerixafor	No	No	✓	✓	✓	×	✓	✓	×	✓	✓	×
Nexavar	Oncological	Sorafenib	No	No	✓	✓	✓	✓	✓	×	✓	✓	×	✓
NexoBrid	Other	Concentrate of proteolytic enzymes enriched in bromelain	No	No	×	×	×	×	×	×	×	×	×	×
Ninlaro	Oncological	Ixazomib	Yes	No	×	×	×	×	×	×	×	×	×	×
Nplate	Other	Romiplostim	No	No	✓	✓	✓	×	✓	✓	×	×	✓	✓
Ocaliva	Other	Obeticholic acid	Yes	No	×	×	×	×	×	×	×	×	×	×
Ofev	Other	Nintedanib	No	No	×	×	✓	✓	✓	×	✓	×	×	×
Onivyde	Oncological	Irinotecan hydrochloride trihydrate	No	No	×	×	×	×	×	×	×	×	×	×
Opsumit	Other	Macitentan	No	No	×	×	×	×	✓	×	×	✓	×	✓
Orphacol	Metabolic	Cholic acid	No	Yes	×	×	×	×	×	×	×	×	×	×
Peyona (previously Nymusa)	Other	Caffeine	No	No	✓	×	×	×	×	×	×	×	✓	×
Plenadren	Metabolic	Hydrocortisone	No	No	×	×	×	×	×	×	×	×	×	×
Procysbi	Other	Mercaptamine	No	No	×	×	×	×	×	×	×	×	×	×
Ravicti	Metabolic	Glycerol phenylbutyrate	No	No	×	×	×	×	×	×	×	×	×	×
Raxone	Other	Idebenone	No	Yes	×	×	×	✓	×	×	×	×	×	×
Revestive	Other	Teduglutide	No	No	×	×	×	×	×	×	×	×	×	×
Revlimid	Oncological	Lenalidomide	No	No	×	✓	✓	✓	✓	×	✓	✓	×	✓
Scenesse	Metabolic	Afamelanotide	No	Yes	×	×	×	×	×	×	×	×	×	×
Signifor	Metabolic	Pasireotide	No	No	✓	×	×	×	×	×	×	×	✓	✓
Siklos	Other	Hydroxycarbamide	No	No	×	×	×	×	×	×	×	×	×	×
Sirturo	Other	Bedaquiline	Yes	No	✓	×	×	×	×	×	×	×	×	×
Soliris	Other	Eculizumab	No	No	×	×	×	×	×	×	×	×	×	×
Sprycel	Oncological	Dasatinib	No	No	✓	✓	✓	✓	✓	✓	✓	✓	✓	✓
Strensiq	Metabolic	Asfotase alfa	No	Yes	×	×	×	×	×	×	×	×	×	×
Strimvelis	Other	Autologous CD34+ enriched cell fraction that contains CD34+ cells transduced with retroviral vector that encodes for the human ADA cDNA sequence	No	No	×	×	×	×	×	×	×	×	×	×
Sylvant	Other	Siltuximab	No	No	×	×	×	×	×	×	×	×	×	×
Tasigna	Oncological	Nilotinib	No	No	✓	×	✓	✓	✓	✓	✓	✓	✓	✓
Tepadina	Oncological	Thiotepa	No	No	×	×	×	×	×	×	×	✓	×	×
Thalidomide Celgene (previously Thalidomide Pharmion)	Oncological	Thalidomide	No	No	×	×	×	✓	✓	×	✓	×	✓	×
Tobi Podhaler	Other	Tobramycin	No	No	✓	×	✓	✓	×	×	✓	×	×	✓
Torisel	Oncological	Temsirolimus	No	No	✓	×	✓	×	✓	×	×	✓	×	✓
Translarna	Other	Ataluren	Yes	No	×	×	×	×	×	×	×	×	×	×
Unituxin	Oncological	Dinutuximab	No	No	×	×	×	×	×	×	×	×	×	×
Venclyxto	Oncological	Venetoclax	Yes	No	×	×	×	×	×	×	×	×	×	×
Vidaza	Oncological	Azacitidine	No	No	✓	×	✓	✓	×	×	✓	✓	×	✓
Vimizim	Metabolic	Elosulfase alfa	No	No	×	×	×	×	×	×	×	×	×	×
Volibris	Other	Ambrisentan	No	No	✓	×	✓	✓	✓	×	✓	✓	✓	✓
Votubia	Oncological	Everolimus	No	No	✓	×	×	✓	×	×	✓	✓	✓	×
Vpriv	Metabolic	Velaglucerase alfa	No	No	×	×	✓	×	×	×	×	✓	×	✓
Vyndaqel	Other	Tafamidis	No	Yes	✓	×	×	×	×	×	×	×	✓	×
Wakix	Other	Pitolisant	No	No	×	×	×	×	×	×	×	×	×	×
Xagrid	Other	Anagrelide	No	Yes	×	×	×	✓	×	×	×	×	×	×
Xaluprine (previously Mercaptopurine Nova Laboratories)	Oncological	Mercaptopurine	No	No	✓	×	✓	✓	✓	×	×	×	×	×
Yondelis	Oncological	Trabectedin	No	No	×	×	×	×	×	×	×	✓	✓	×
Zalmoxis	Oncological	Allogeneic T cells genetically modified with a retroviral vector encoding for a truncated form of the human low affinity nerve growth factor receptor (LNGFR) and the herpes simplex I virus thymidine kinase (HSV-TK Mut2)	Yes	No	×	×	×	×	×	×	×	×	×	×
Zavesca	Metabolic	Miglustat	No	No	✓	×	✓	×	×	×	×	×	×	✓

*✓ – reimbursed; × – not reimbursed.*

### Comparative Summary of Orphan Drug Reimbursement Policy

Half of analyzed countries implemented special regulations regarding (including different sources of payment or relaxed reimbursement requirements like higher thresholds) the reimbursement of orphan drugs. In most countries, the marketing authorization holder (MAH) applies for reimbursement of a particular drug; however, in Estonia and Lithuania, applications by doctors’ or patients’ organizations are also possible. Reimbursement decisions are made mostly by bodies responsible for health policies in individual countries, e.g., ministries of health (MoH). In some countries, other bodies are also included in the reimbursement decision-making process. In all analyzed countries the list of reimbursed drugs is publicly available (Table 2).

**Table 2:** Summary of reimbursement aspects in Central Eastern European countries.

Question	Bulgaria	Croatia	Czechia	Estonia	Hungary	Latvia	Lithuania	Poland	Romania	Slovakia
Are there any special laws /policies regarding orphan drugs different from the ones for non-orphan drugs?	No	Yes	No	No	Yes	No	Yes^∗^	No	Yes	Yes
If yes, could you please describe in what way the special law/policy for orphan drugs differ from that for non-orphan drugs?	NA	Source of payment	NA	NA	e.g., acceptability of cost-effectiveness	NA	Positive decision of The Ultra-rare diseases reimbursement Committee	NA	Special therapeutic programs	No QALY thresholds
Who (or what entity) provides reimbursement decisions (decisions on the coverage of a part or whole cost of orphan drugs from public budget)?	The National Council for Pricing and Reimbursement of Medicinal Products	CHIF	SUKL	Ministry of Social Affairs, Health Insurance Fund	NIHIFM, Department of HTA, HTA Committee, Ministry of Human Capacities	The National Health Service of Latvia or Committee	Reimbursement committee or Ultra-rare diseases reimbursement Committee	MoH	Drug Agency evaluation, MoH, NHIH	MoH
Is the list of reimbursed drugs publicly available?	Yes	Yes	Yes	Yes	Yes	Yes	Yes	Yes	Yes	Yes
Who applies for reimbursement? Is it the MAH or a public decision-making body, e.g., the MoH?	MAH	MAH	MAH, importer, domestic manufacturer, health insurance company	MAH, doctors’ organizations	MAH	MAH, authorized representative, wholesaler	MAH, doctors’ or patients’ organizations, doctor application for an individual patient (ultra-rare disease)	MAH	MAH	MAH

*MAH, Marketing Authorization Holder; MoH, Ministry of Health; QALY, Quality-Adjusted Life Year; ICER, Incremental Cost-Effectiveness Ratio; GDP, Gross Domestic Product; CEA, Cost-Effectiveness Analysis; CUA, Cost-Utility Analysis; CMA, Cost-Minimization Analysis; BIA, Budget Impact Analysis; NA, Not Applicable; HTA, Health Technology Assessment; SUKL, State Institute for Drug Control in the Czechia; NHIH, National Health Insurance House in Romania; CHIF, Committee for Medicines in Croatia; NIHIFM, National Institute of Health Insurance Fund Management. “^∗^” indicates ultra-orphan (1:200,000) diseases.*

A full or simplified health technology assessment (HTA) of a submitted application should be performed with reimbursement application in most analyzed countries except Romania and Lithuania. In Romania, the system is based on score cards that consider the reimbursement status of a particular drug in other countries (United Kingdom, Germany, and France), and in Lithuania there is a formal HTA process implemented but it does not include economic assessment. This is why in some of those countries HTA is limited to the assessment of clinical data in other it also includes analysis of cost-effectiveness, e.g., ICER and/or quality-adjusted life year (QALY; or Life Years Gained – LYG). In all other countries ICER providing information on marginal cost per QALY is employed in reimbursement decision-making, but no higher threshold value is implemented for orphan drugs compared with non-orphan drugs (Table 3).

**Table 3:** Summary of health technology assessment aspects in Central Eastern European countries.

Question	Bulgaria	Croatia	Czechia	Estonia	Hungary	Latvia	Lithuania^∗^	Poland	Romania	Slovakia
What HTA analyses should be provided during the reimbursement application process (e.g., cost-effectiveness analysis, budget impact analysis, clinical analysis, etc.)?	Clinical analysis, CEA/CUA, BIA, ethical considerations.	BIA, CEA, clinical analysis, expert opinion	BIA, CEA/CUA, clinical analysis	Full HTA dossier required for orphan drugs	Full economic evaluation (CEA/CUA, BIA)	Clinical analysis, price analysis, CEA/CMA, BIA	No formal HTA process; however, clinical analysis, CEA/CUA, BIA should be provided.	CEA/CUA/CMA, BIA, clinical analysis, rationalization analysis	Score cards	Clinical analysis, CEA/CUA, BIA
Is ICER value important in reimbursement decision-making?	Yes	Yes	Yes	Yes.	Yes	Yes	NA	Yes	No	Yes
Are there any thresholds for ICER valid in reimbursement decision-making in your country?	No	Yes	No	No	Yes	Yes	NA	Yes	No	Yes
Is there another (higher) ICER threshold for orphan drugs?	No	No	No	No	No	No	NA	No	No	NA

*MAH, Marketing Authorization Holder; MoH, Ministry of Health; QALY, Quality-Adjusted Life Year; ICER, Incremental Cost-Effectiveness Ratio; GDP, Gross Domestic Product; CEA, Cost-Effectiveness Analysis; CUA, Cost-Utility Analysis; CMA, Cost-Minimization Analysis; BIA, Budget Impact Analysis; NA, Not Applicable; HTA, Health Technology Assessment; SUKL, State Institute for Drug Control in the Czechia; NHIH, National Health Insurance House in Romania; CHIF, Committee for Medicines in Croatia; NIHIFM, National Institute of Health Insurance Fund Management. “^∗^” indicates ultra-orphan (1:200,000) diseases.*

### The Agreement in Reimbursement Decisions

The highest agreement in reimbursement decisions was observed between Estonia and Lithuania, and the lowest – between Estonia and Latvia, with kappa coefficients of 0.69 and 0.11, respectively. In all pairwise comparisons the agreement was higher than 0; however, in a few pairs the lower bound of the confidence interval was negative, which indicated that there was no observed agreement in reimbursement decisions between those countries (Czechia and Romania, Estonia and Latvia, Estonia and Romania, Hungary and Romania, Romania and Slovakia; Table 4).

**Table 4:** Kappa coefficients of agreement in reimbursement status of analyzed drugs among Central Eastern European countries.

Country	Croatia	Czechia	Estonia	Hungary	Latvia	Lithuania	Poland	Romania	Slovakia
Bulgaria	0.42 (0.21–0.64)	0.37 (0.16–0.58)	0.39 (0.18–0.60)	0.41 (0.20–0.62)	0.26 (0.05–0.46)	0.31 (0.09–0.53)	0.40 (0.20–0.61)	0.51 (0.30–0.71)	0.49 (0.28–0.70)
Croatia		0.27 (0.06–0.48)	0.22 (0.00–0.43)	0.43 (0.20–0.66)	0.55 (0.26–0.84)	0.27 (0.02–0.52)	0.39 (0.18–0.59)	0.26 (0.03–0.49)	0.30 (0.06–0.54)
Czechia			0.42 (0.22–0.63)	0.51 (0.30–0.71)	0.25 (0.05–0.44)	0.48 (0.27–0.69)	0.49 (0.29–0.69)	0.20 (−0.02–0.41)	0.53 (0.33–0.73)
Estonia				0.41 (0.20–0.62)	**0.11 (−0.07**–**0.29)**	**0.69 (0.51**–**0.86)**	0.40 (0.20–0.61)	0.21 (−0.00–0.43)	0.25 (0.03–0.47)
Hungary					0.40 (0.17–0.64)	0.45 (0.23–0.68)	0.54 (0.35–0.74)	0.22 (−0.00–0.45)	0.45 (0.23–0.67)
Latvia						0.20 (−0.05–0.45)	0.23 (0.04–0.42)	0.30 (0.08–0.53)	0.25 (0.01–0.48)
Lithuania							0.52 (0.32–0.72)	0.23 (0.00–0.46)	0.41 (0.17–0.64)
Poland								0.35 (0.14–0.56)	0.34 (0.12–0.55)
Romania									0.18 (−0.05–0.40)

*The highest and lowest agreements are marked bold.*

### Reimbursement Decisions in the Context of the Type of Authorization and Disease

In total, 14 drugs (14.74%) were approved conditionally and another 14 drugs (14.74%) were approved under exceptional circumstances. The type of authorization was associated with the type of disease (*p*-value of 0.0053). Medicinal products for the treatment of genetic metabolic disorders were usually authorized under exceptional circumstances, and oncological drugs – under conditional approval (Table 5).

**Table 5:** Relationship between type of the disease and type of approval in European Union.

Disease type	Conditional Approval	Exceptional Circumstances	Unconditional	Total	*p*-value (FET)
Metabolic	0 (0.00%)	7 (31.82%)	15 (68.18%)	22	0.0053^∗^
Oncological	9 (25.00%)	2 (5.56%)	25 (69.44%)	36	
Other	5 (13.51%)	5 (13.51%)	27 (72.97%)	37	
Total	14	14	67	95	

*p-value less than 0.05 is marked with an asterisk. FET, Fisher Exact Test.*

The reimbursement status was significantly associated with the type of approval only in the Czechia, Lithuania, and Slovakia. In those countries no drugs approved conditionally were reimbursed (out of all analyzed drugs – other conditionally approved drugs could be reimbursed like Erivedge for advanced basal cell carcinoma was in The Czechia); however, in other countries at least one drug that was conditionally approved was reimbursed. In Lithuania, Slovakia, and Latvia, no drugs approved under exceptional circumstances were reimbursed – however, they may be available on patient bases, because of rarity of the disorder (Table 6).

**Table 6:** Relationship between reimbursement status and type of approval in Central Eastern European countries.

Country	Reimbursement status	Conditional Approval	Exceptional Circumstances	Unconditional	Total	*p*-value
Bulgaria	Reimbursed	3 (12.50%)	3 (12.50%)	18 (75.00%)	24	0.8568
	Not reimbursed	11 (15.49%)	11 (15.49%)	49 (69.01%)	71	
Croatia	Reimbursed	2 (18.18%)	1 (9.09%)	8 (72.73%)	11	0.8279
	Not reimbursed	12 (14.29%)	13 (15.48%)	59 (70.24%)	84	
Czechia	Reimbursed	0 (0.00%)	2 (8.00%)	23 (92.00%)	25	0.0161^∗^
	Not reimbursed	14 (20.00%)	12 (17.14%)	44 (62.86%)	70	
Estonia	Reimbursed	2 (8.33%)	2 (8.33%)	20 (83.33%)	24	0.2817
	Not reimbursed	12 (16.90%)	12 (16.90%)	47 (66.20%)	71	
Hungary	Reimbursed	2 (10.00%)	1 (5.00%)	17 (85.00%)	20	0.2506
	Not reimbursed	12 (16.00%)	13 (17.33%)	50 (66.67%)	75	
Latvia	Reimbursed	1 (16.67%)	0 (0.00%)	5 (83.33%)	6	0.5744
	Not reimbursed	13 (14.61%)	14 (15.73%)	62 (69.66%)	89	
Lithuania	Reimbursed	0 (0.00%)	0 (0.00%)	16 (100.00%)	16	0.0179^∗^
	Not reimbursed	14 (17.72%)	14 (17.72%)	51 (64.56%)	79	
Poland	Reimbursed	2 (7.69%)	3 (11.54%)	21 (80.77%)	26	0.3704
	Not reimbursed	12 (17.39%)	11 (15.94%)	46 (66.67%)	69	
Romania	Reimbursed	1 (4.76%)	3 (14.29%)	17 (80.95%)	21	0.3264
	Not reimbursed	13 (17.57%)	11 (14.86%)	50 (67.57%)	74	
Slovakia	Reimbursed	0 (0.00%)	0 (0.00%)	19 (100.00%)	19	0.0070^∗^
	Not reimbursed	14 (18.42%)	14 (18.42%)	48 (63.16%)	76	
Total		14	14	67	95	

*p-values less than 0.05 are marked with an asterisk.*

The relationship between the type of disease and the reimbursement status was significant in Croatia, Estonia, Hungary, and Lithuania (Table 7). In all those countries, most reimbursed drugs were indicated for the treatment of oncological diseases. Logistic regression supports these results and in Croatia, oncological orphan drugs were more than five times more likely to be reimbursed compared with the remaining drugs (OR of 5.33; 95%CI: 1.31–21.68; *p*-value of 0.0124). In Estonia the odds for reimbursement were 90% lower for metabolic than for other orphan drugs (OR of 0.10; 95%CI: 0.01–0.82; *p*-value of 0.0314).

**Table 7:** Relationship between reimbursement status and type of rare disease in analyzed Central Eastern European countries.

Country	Reimbursement status	Metabolic	Oncological	Other	Total	*p*-value
Bulgaria	Reimbursed	4 (16.67%)	13 (54.17%)	7 (29.17%)	24	0.1639
	Not reimbursed	18 (25.35%)	23 (32.39%)	30 (42.25%)	71	
Croatia	Reimbursed	1 (9.09%)	8 (72.73%)	2 (18.18%)	11	0.0403^∗^
	Not reimbursed	21 (25.00%)	28 (33.33%)	35 (41.67%)	84	
Czechia	Reimbursed	5 (20.00%)	10 (40.00%)	10 (40.00%)	25	0.9069
	Not reimbursed	17 (24.29%)	26 (37.14%)	27 (38.57%)	70	
Estonia	Reimbursed	1 (4.17%)	13 (54.17%)	10 (41.67%)	24	0.0259^∗^
	Not reimbursed	21 (29.58%)	23 (32.39%)	27 (38.03%)	71	
Hungary	Reimbursed	0 (0.00%)	12 (60.00%)	8 (40.00%)	20	0.0104^∗^
	Not reimbursed	22 (29.33%)	24 (32.00%)	29 (38.67%)	75	
Latvia	Reimbursed	0 (0.00%)	4 (66.67%)	2 (33.33%)	6	0.2306
	Not reimbursed	22 (24.72%)	32 (35.96%)	35 (39.33%)	89	
Lithuania	Reimbursed	0 (0.00%)	10 (62.50%)	6 (37.50%)	16	0.0231^∗^
	Not reimbursed	22 (27.85%)	26 (32.91%)	31 (39.24%)	79	
Poland	Reimbursed	4 (15.38	15 (57.69	7 (26.92	26	0.0507
	Not reimbursed	18 (26.09%)	21 (30.43%)	30 (43.48%)	69	
Romania	Reimbursed	5 (23.81%)	9 (42.86%)	7 (33.33%)	21	0.8194
	Not reimbursed	17 (22.97%)	27 (36.49%)	30 (40.54%)	74	
Slovakia	Reimbursed	4 (21.05%)	10 (52.63%)	5 (26.32%)	19	0.3043
	Not reimbursed	18 (23.68%)	26 (34.21%)	32 (42.11%)	76	
Total		22	36	37	95	

*p-values less than 0.05 are marked with an asterisk.*

### Reimbursement Policy in Analyzed Countries

In Bulgaria, the reimbursement requirements for orphan drugs are the same as for other medicinal products; the initiative for reimbursement is only by MAH. The National Council on Prices and Reimbursement of Medicinal Products is responsible for the final decision about the reimbursement and the level of reimbursement. Most orphan drugs are paid from the budget of the National Health Insurance Fund, but some are paid from hospital budgets. Orphan drugs need to be included in Annex I (or Annex II) of the publicly available Positive Drug List. The reimbursement level depends on the type of disease, type of treatment (essential, symptomatic, palliative, or other), and budget resources allocated for procurement of the medicinal product. The level or reimbursement for orphan drugs is usually 100% or, in some rare cases, 75%. In the process of reimbursement, the decision-maker performs an additional assessment based on the severity of a rare condition, the availability of an alternative product, and the cost for the patient if the medicinal product is not reimbursed. The process also considers if the drug has an orphan status which mean the drug has a great social benefit and their use is indicated for serious conditions for which there is no effective alternative therapy. For Bulgaria, the ICER value is not published in normative documents such as regulations and law. The National Council on prices and reimbursement has published methodological recommendations on documentation presented for assessment of the efficacy, safety and pharmacoeconomic parameters of medicinal products applying for inclusion in the Positive Drug List. The pharmacoeconomic analysis shall indicate whether the medicinal product is cost-effective using the World Health Organization’s CHOICE programme (Choosing Interventions that are Cost-Effective). The result must be presented as GDP in Bulgarian currency and in the purchasing power standard (adjusted by the purchasing power parity), according to official data published by the National Statistical Institute (Tables 2, 3).

In Croatia, drugs for rare diseases are delivered through hospitals. There is a special fund (part of the Croatian Health Insurance Fund [CHIF]) for orphan drugs. The reimbursement of these drugs will not burden a hospital budget. Orphan drugs included in the essential list of drugs of the CHIF are completely reimbursed, while those included in the additional list are partially reimbursed. During the reimbursement application process, a budget impact analysis should be provided. A cost-effectiveness analysis, a clinical analysis, or an expert opinion can be additionally provided if needed. The ICER value is important or even essential in the reimbursement decision; in some cases, there is a need to provide additional budget impact analysis results (Tables 2, 3).

In The Czechia, the State Institute for Drug Control (a governmental regulatory agency) decides on pricing and reimbursement of drugs used in outpatient (ambulatory) care. However, SUKL does not decide on drugs used in in-patient (hospital) care. These drugs are reimbursed from hospital budgets (based on agreements between hospitals and health insurance funds). In the context of orphan medicines, it is important to note that if any medicine is not approved to be reimbursed from any reason, it can still be reimbursed on individual patient request if this is the only treatment available for the individual patient taking account of his/her clinical state. In this case, the reimbursement of this medicine needs to be pre-approved by the patient’s health insurance fund. Several entities could apply for reimbursement: a MAH (for authorized medicinal products), an importer or domestic manufacturer (for foods for special medical purposes or non-registered medical products used in the Czechia within a specific treatment program), and health insurance companies. The budget impact analysis is required in the HTA process, and the cost-effectiveness analysis is required in most cases. In some cases, a similar efficacy and safety profile of applicant drug in relation to comparators make a cost-minimization analysis possible. HTA analyses are required in all cases when reimbursement conditions are broadened (such as new indications, fewer restrictions on target patient groups) in comparison with the current state or a therapeutically interchangeable intervention. The ICER value is important in decision-making except for temporary reimbursement applications granted for a minimum of 2 years, renewable for another year. Usually, medicines with temporary reimbursement are highly innovative products (i.e., new medicines for very serious diseases with an unmet medical need). The current legislation does not define a threshold for ICER. However, during the HTA process, the State Institute for Drug Control compares the ICER of the assessed technology with the ICERs of already reimbursed technologies (used for similar indications or similar patient groups). The usual ICER used in line with the institute’s decision-making practice is 1.2 million CZK per QALY (about 44,500 EUR per QALY; Tables 2, 3).

Estonia has not implemented special reimbursement legislation for orphan drugs. For all drugs reimbursement decisions are performed by the Ministry of Social Affairs and the funding is provided by the Health Insurance Fund. Only MAH or doctors’ organizations can apply for reimbursement, and a full HTA dossier is required for orphan drugs. However, ICER is important in the decisions-making process not specific threshold are defined neither for orphan nor non-orphan drugs.

In Hungary, there is no separate legislation for orphan drugs; however, some policies apply particularly to this drug class, such as acceptability of cost-effectiveness or importance of the role of equity. One of the entities that can apply for the reimbursement of an orphan drug is an HTA committee established by the National Institute of Health Insurance Fund Management. The other route is through the Ministry of Human Capacities: the National Institute sends a recommendation on drug reimbursement to the Ministry; for new active substances, it is necessary to amend the law for the reimbursement (e.g., in the case of a new indication). The Ministry is in charge of the amendment. In most cases, the representative of the MAH, but sometimes the MAH itself, applies for a reimbursement dossier. The value of ICER is a very important criterion, but there are more criteria that must be considered such as equity, budget impact, or the disease severity. The ICER cannot be higher than 3 times the GDP per capita; however, there is no separate threshold for orphan drugs (Tables 2, 3).

Latvia does not have any special legislation regarding orphan drugs. Orphan medicines are partially available via the positive reimbursement list; some orphans are available as a part of the special programme of rare diseases for Children’s University Hospital, Riga. Some orphan drugs are provided within individual reimbursement with limitation up to 14,228.72 euro/year for a single patient. The reimbursement process is started by the holder of a registration certificate, an authorized representative, or a wholesaler by submitting by submitting a full dossier. If orphan drugs are submitted to be reimbursement in the Positive list or individual reimbursement the decision is made by The National Health Service of Latvia. If an orphan drug is used to treat the very rare disease the decision is made by the Committee. In all cases, the applicant should provide clinical, cost-effectiveness, and the budget impact analyses. The ICER value is important in the decision-making process. The calculation of the costs for one unit of an additionally obtained result of therapeutic efficacy (ICER), the coefficient of expansion of cost-effectiveness for a life-year gained or a progression-free survival do not exceed the three times the GDP per capita. The economic analysis also takes into account the proof of the cost-effectiveness of the medicinal products in the health care system at large or for a specific group of patients (Tables 2, 3).

Lithuania implemented separate legislation for orphan drugs for very rare diseases. Only a drug used for an ultra-rare disease (defined in Lithuania as a disease or human health condition with one newly diagnosed case per 200,000 inhabitants per year) can be reimbursed. If orphan drugs are applicable to be reimbursement in the Positive list the decision is made by the Reimbursement committee. If an orphan drug is used to treat the ultra-rare disease the decision is made by The Ultra-rare diseases reimbursement Committee according to the doctor’s application. The MAH, as well as doctors’ or patients’ organizations, could apply for reimbursement of orphan drugs. However, in the case of drugs for ultra-rare diseases, only the doctor’s application for reimbursement for an individual patient is acceptable. Reimbursement may depend on the prevalence of the disease (orphan drug vs. oncology drug) and on the application (there may be that the MAH has not applied for reimbursement). The HTA process in Lithuania is not implemented yet, but in its application for a drug to be included in the positive list, the MAH should provide clinical, cost-effectiveness, and the budget impact analyses. If doctors apply to the Committee for reimbursement of drugs used for ultra-rare disease, they should provide information on the patient’s clinical condition and substantiation of orphan drug use (Tables 2, 3).

Poland does not implement any separate legislation for orphan drugs, which are treated as ordinary medications. However, such drugs could be reimbursed for individual patients. If it is the case an approval is granted by the Ministry of Health, a drug is financed from a hospital budget. Multi-criteria decision analysis (MCDA) is considered as a strategic direction indicating an additional element in the decision-making process for orphan drugs reimbursement in Poland (Ministry of Health, 2019). The key policy maker and the regulator in the health care system in Poland is the Ministry of Health, supported by advisory bodies. The AOTMiT is an independent legal entity that collects data and delivers statements and recommendations on technologies claiming public funding, of which predominant are drugs. The Transparency Council, which is an independent advisory body consisting of 20 highly qualified members providing opinions for applicant drugs. The final reimbursement decisions are taken independently by the Minister of Health, and the decisions do not have to comply with statements or recommendations issued by the Transparency Council or the President of the AOTMiT. Poland implements external reference pricing, internal reference pricing, value-based pricing and negotiations when establishing price of drugs (Tables 2, 3).

The case of Romania is fundamentally different, orphan drugs being included in a therapeutic program for the rare disease. MAH submits the file of the product to the National Drug and Medical Device Agency (NDMDA). The evaluation consists mainly of allocating to every drug several points for its reimbursement status in the United Kingdom, Germany, and France. Orphan drugs are receiving a bonus score comparative with other molecules. This is so called Score cards method. The Government approves the NDMDA’s recommendation through a Government Decision published in the *Official Gazette of Romania*. After issuing a therapeutic protocol for the new drug the reimbursed status becomes effective by the National Health Insurance House (NHIH) and MoH jointly order (Tables 2, 3).

Slovakian reimbursement decisions were in 2017 based on thresholds (commonly described with the Greek letter “λ”) set forth by Act No. 363/2011 Z. z. The lower threshold (λ1) was defined as 24 times the average monthly salary (21,192 EUR/QALY), and the upper threshold (λ2), as 35 times the average monthly salary (30.905 EUR/QALY). The medicine was reimbursed from public health insurance (fully or partially) if the incremental costs were lower or equal to λ1 per one QALY. The medicine was conditionally reimbursed if the incremental costs lied within λ1 and λ2 thresholds per one QALY. Medicinal products whose additional costs per QALY exceeded the upper λ2 threshold should not be included in the reimbursement list. These thresholds were not applicable for orphan drugs indicated for therapy of rare diseases with prevalence lower than 1:100,000 in Slovakia.

Based on the new Slovak legislation (updated Act No. 363/2011 Z. z.), which came into the force in January 1st 2018, Slovakian reimbursement decisions was in 2018 based on the following thresholds:

• lower threshold (λ1): 35 times average monthly salary (total 31.920 EUR/QALY);
• upper threshold (λ2): 41 times average monthly salary (total 37.392 EUR/QALY).

In general, the medicine is reimbursed from public health insurance (fully or partially) if the incremental costs were lower or equal to λ1 per one incremental QALY. In defined cases could be the thresholds per one incremental QALY increased up to λ2.

Based on the Slovak legislation, which came into the force in January 1, 2018, the cost – effectiveness thresholds were not used in 2018 for medicines in the following cases: an applicant do not need to attach a pharmacoeconomic analysis for the decision making procedure at the Slovak Ministry of Health concerning to reimbursement from the public health fund in the case that a medicinal product is aimed for treatment of disease, for which the number of patients eligible for treatment with the medicinal product based on the indication approved in marketing authorization was in the Slovak republic lower than 1: 50,000.

The required dossiers obligatory in reimbursement procedures should be submitted by the MAH and have to include basic drug information, evidence on its effectiveness, the standard therapeutic dose, and the number of standard therapeutic doses per package. Applications also contain the proposed reimbursement rate, indication, and restriction of prescription and/or indication, if applicable.

After a medicine receives market authorization, the Ministry of Health of the Slovak Republic determines its maximum retail price (ex-factory price), applying external reference pricing methodology. The final price of each medicine available on the Slovak pharmaceutical market may not exceed the average of the three lowest prices of the same medicine available on pharmaceutical markets across the EU. The Slovak Ministry of Health established the Reimbursement (or Categorization) Committee to act as its advisory body on reimbursement processes. The Committee prepares recommendations for reimbursement levels, patients’ co-payments, and conditions for reimbursement. The decision about the reimbursement levels of eligible medicines is based on the following criteria: therapeutic benefit of the medicine; cost-effectiveness; and the reimbursed levels of other medicines within the same reference group. The final reimbursement (or categorization) list also includes medicines with prescription or indication restrictions. In the case of certain oncological medicines, the reimbursement can also be restricted to prescription solely in specialized hospitals. Based on the recommendations from the Categorization Committee the Ministry of Health issues final decisions (Tables 2, 3).

## Discussion

The objective of this study was to provide a comprehensive description of orphan drug reimbursement policies in EU–CEE countries. Moreover, we aimed to assess the agreement in reimbursement decisions between those countries as well as the impact of the type of approval and disease (oncological or metabolic) on reimbursement decisions. We observed that half of the analyzed countries imposed specific regulations regarding reimbursement of orphan drugs; however, none of the countries used higher an ICER threshold (marginal costs per QALY) for orphan drugs. The share of reimbursed orphan drugs varied significantly across the countries; however, it was not significantly associated with neither GDP not GDP per capita. The agreement between the countries varied from slight agreement (Estonia vs. Latvia) to substantial agreement (Estonia vs. Lithuania); however, the agreement was also affected by the different shares of reimbursed orphan drugs. Our study revealed that there are differences in reimbursement and HTA policies across so called Baltic countries. In Lithuania no formal HTA process has been implemented; in Latvia no special laws or policies regarding orphan drugs different from the ones for non-orphan drugs are in force; in Lithuania the special policies apply only to ultra-orphan drugs (defined as indicated for illnesses with a prevalence of 1:200,000 or lower). The differences between those countries could be also noticed from the perspective of burden of healthcare on households’ budgets. The financial burden of paying for medicines in 2017 in EU countries varied significantly with Estonia being one of the countries with the smallest share of households with high burden and Latvia and Lithuania being one of the countries with the highest share of households with high burden (European Commission, 2019).

In The Czechia, Lithuania and Slovakia, the reimbursement statuses were significantly associated with the type of approval; while in Croatia, Estonia, Hungary and Lithuania, the type of disease was significantly associated with the reimbursement status. In all those countries, most reimbursed drugs were indicated for the treatment of oncological diseases.

In some countries limitations in reimbursement only for some subgroups of patients due to budgetary constraints are applicable; public coverage is limited only for patients fulfilling some inclusion criteria e.g., stage or severity of disease.

To compare the results of our study with current knowledge on the subject, we performed a systematic review of publications in medical databases. We identified a study from 2016 by Zelei et al. (2016), who reviewed scientific evidence on the HTA of orphan drugs with a special focus on public payers in CEE countries. The authors observed that only 5 of 87 publications included in the analysis referred to CEE countries, which indicates the need for further research. As CEE countries are more budget-restricted than western countries, they could be more affected by the lack of clinical evidence for orphan drugs, which generally gain marketing authorization earlier than non-orphan drugs. Our present study showed that the type of marketing authorization plays an important role in many CEE countries. If the accessibility of orphan drugs remains at the same level in the CEE region as in western EU countries, the relative budget impact could be significantly higher.

The study from 2012 by Iskrov et al. (2012) focused on the perspective on Bulgaria in terms of reimbursement of orphan drugs. The authors revealed that of all 61 orphan drugs approved in the EU in 2011, only 16 were available in Bulgaria and the mean waiting time for reimbursement decision was 43 months (standard deviation, 29 months). Similarly, to our study, the author emphasized the need for special legislation for orphan drugs that are not only based on epidemiological but, more importantly, on economic factors for better assets allocation.

Pavlović et al. (2012) revealed that in 2012 in Bulgaria the Positive Drug List included 44.3% (27 out of 61) of the drugs with prior orphan designation, as compared with only 25% (17 out of 68) in Serbia and 52.5% (32 out of 61) in Sweden, which also indicated a difference between Eastern and Western part of Europe.

Logviss et al. (2014) evaluated a situation in Latvia in 2014. They revealed that 34 orphan drugs were available in Latvia, although only three were reimbursed (all indicated for Philadelphia chromosome–positive chronic myeloid leukemia). Additionally, 15 drugs (44.1%) were reimbursed for individual patients and another five drugs (14.7%) were reimbursed as part of a medical treatment program for rare diseases in children.

Picavet et al. (2012) analyzed access to orphan drugs for almost all EU countries (except for Cyprus, Malta, and Portugal) based on data from IMS Health (2011). They showed that employing an HTA process plays an important role in the patients’ access to reimbursed orphan drugs, which mostly affect low-GDP countries. However, nowadays more low-GDP countries use a formal HTA process than in 2011.

Gammie et al. (2015) analyzed regulations and policies used by countries to allow patient access to orphan drugs in 2015 by performing a systematic review of evidence published between 1998 and 2014. They summarized legislations of 35 countries from around the world, including 21 from the EU, and revealed that a different type of special regulations for orphan drugs (national orphan drug policies, orphan drug designation, marketing authorization, marketing exclusivity, and tax credits) was present in most of the countries. A variation in the share of orphan drugs accessible for the patients was also observed.

Kamusheva et al. (2018) provided a comprehensive description of access of patients with rare diseases to biotechnological drugs in several CEE countries in 2018, showing that special legislation for orphan drugs was implemented in several CEE countries. The share of accessible orphan drugs as well as total expenditures varied across countries, being the highest in Greece and the lowest in Romania.

Szegedi et al. (2018) revealed that from 29.4 to 92.8% of the 83 orphan drugs were available (and reimbursed) in 2015 in 8 EU countries in favor of the higher-income ones. The highest expenditure on orphan drugs in the years from 2013 to 2014 was observed in Belgium (245–280 million Euro) and the lowest in Bulgaria (8.3–12.2 million Euro).

Another study assessed Bulgarian legislation on HTA and reimbursement decision-making criteria, with a special focus on orphan and innovative drugs. A critical analysis of current decision-making criteria for drug reimbursement was performed, and a comprehensive assessment scoring system for orphan drugs with decision-making criteria was scheduled, including the presence of therapeutic alternative, clinical effectiveness, safety, pharmacoeconomics, and societal value, which were divided into weighted indicators. The study revealed that Bulgarian reimbursement decision-making seems not to be sufficiently transparent and not effective in innovative HTA, with access to a therapeutic alternative as a key reimbursement decision-making criterion for orphan drugs (Iskrov et al., 2013).

In the recent study Czech et al. (2018) compared rare disease definitions and epidemiology, diagnostics and new-born screening, national plans, patient registries and reimbursement of orphan drugs including HTA processes in Poland, Russia, and the Netherlands. There are clear differences in healthcare expenditure and rare disease policies between these countries. Access to reimbursed orphan drugs varies widely between these three countries, and sometimes even within (Russia). Budgeting structures (i.e., federal vs. regional) play a large role in regional healthcare access for patients, especially in Russia, where local government institutions and budgets often determine the type and level of healthcare provided. These findings were confirmed in our analyses.

In our previous study (Malinowski et al., 2018) we have analyzed orphan drugs reimbursement policies in selected Western European countries. We have observed that the share of reimbursed orphan drugs is significantly higher in Western Europe than in the CEE states however, the agreement between countries has not present any spatial relationship as in the current study. In both studies we have observed a significant influence of both disease type and EMA drug authorization type on reimbursement decisions in some countries - conditional approval significantly decreased the chance for reimbursement in France, Italy, and Spain by 77–80%; approval granted under exceptional circumstances had significant impact only in Germany with 85% decrease in chances for reimbursement. The different shares of reimbursed drugs between previous and current studies (which is an obvious finding) make comparisons of results of both projects difficult.

Our study is the first to comprehensively analyse of the impact of the type of EMA approval and the type of the disease on orphan drug reimbursement decision-making, which constitutes the major strength of this study. The results should aid orphan drug management and policies in a number of countries, including CEE countries. The current and updated review of reimbursement decisions among countries and international comparisons provide additional input for proper and effective reimbursement decision-making. Moreover, we collected the data in cooperation with a number of local experts familiar with reimbursement policy in each country, so the input is worthwhile and credible. There is an institutional regional cooperation initiative worth mentioning based on a memorandum of understanding signed by selected CEE countries called V4+ Fair and Affordable Pricing. Its ultimate goal is to develop and harmonize methods of cooperation and negotiations with MAHs concerning pricing and conditions for reimbursement of selected health technologies with a special emphasis on the highest priced drugs including orphan medicinal products. The objective is to build an active institutional network, exchange of expertise and experience in pricing and reimbursement and conduct common health technology assessment aimed at facilitation access to effective and affordable treatment solutions. In our study we not only did the regulation analysis but measure the regulatory agreement, as well as also try to find its possible correlation with other factors as GDP of the countries. We have applied κ coefficient to measure the extent of agreement between countries that is above the random (at chance). In addition, we used logistic regression to calculate odds for positive reimbursement decision in association with the type of the disease. Using those statistical methods is inevitable strength of the study.

Our study has also some limitations. First of all, we analyzed drugs with orphan designations granted in 2017. We also collected data valid for 2017 due to changes in reimbursement systems in included countries, so our results will need an update in the coming years. Moreover, a constant monitoring of reimbursement statuses in analyzed countries, with conclusions on current trends in reimbursement decision-making for orphan drugs would be especially beneficial - so the issue needs further assessment and additional studies. Chances for reimbursement in analyzed countries could be also affected by the prevalence of the diseases, which should be tested during further studies.

## Conclusion

The study revealed that some of the considered countries already established separate regulations on reimbursement of orphan drugs; in case of some of these countries higher ICER values for orphans are used; in Lithuania and Romania, no formal HTA process was employed. The share of reimbursed orphan drugs varied significantly across the countries, but it was not associated either with GDP or GDP per capita. The lowest (slight) agreement in reimbursement decisions was observed between Estonia and Latvia, and the highest (substantial) agreement, between Estonia and Lithuania. In The Czechia, Lithuania and Slovakia, EMA’s conditional approval significantly decreased the chances for reimbursement. In Croatia, Estonia, Hungary, and Lithuania, drugs for oncological diseases had significantly greater chances for reimbursement.

## Data Availability

The datasets generated for this study are available on request to the corresponding author.

## Author Contributions

KM performed the data synthesis and analysis as well as drafted the manuscript. PK designed and supervised the study, and wrote the final manuscript. WT supervised the study and critically reviewed the manuscript. MC contributed to review the manuscript and wrote the final version of the manuscript. GP, MM, AS, PD, LV, JS, AM, KM, ZR, JG, TT, and MP provided necessary data and wrote country-specific sections of the manuscript.

## Conflict of Interest Statement

The authors declare that the research was conducted in the absence of any commercial or financial relationships that could be construed as a potential conflict of interest.
